# Free energy landscape of G-protein coupled receptors, explored by accelerated molecular dynamics†
†Electronic supplementary information (ESI) available. See DOI: 10.1039/c3cp53962h



**DOI:** 10.1039/c3cp53962h

**Published:** 2014-01-21

**Authors:** Yinglong Miao, Sara E. Nichols, J. Andrew McCammon

**Affiliations:** a Howard Hughes Medical Institute , University of California at San Diego , La Jolla , CA 92093 , USA . Email: yimiao@ucsd.edu; b Department of Chemistry and Biochemistry , University of California at San Diego , La Jolla , CA 92093 , USA . Email: senichols@ucsd.edu; c Department of Pharmacology , University of California at San Diego , La Jolla , CA 92093 , USA

## Abstract

G-protein coupled receptors (GPCRs) mediate cellular responses to various hormones and neurotransmitters and are important targets for treating a wide spectrum of diseases. They are known to adopt multiple conformational states (*e.g.*, inactive, intermediate and active) during their modulation of various cell signaling pathways. Here, the free energy landscape of GPCRs is explored using accelerated molecular dynamics (aMD) simulations as demonstrated on the M2 muscarinic receptor, a key GPCR that regulates human heart rate and contractile forces of cardiomyocytes. Free energy profiles of important structural motifs that undergo conformational transitions upon GPCR activation and allosteric signaling are analyzed in detail, including the Arg^3.50^–Glu^6.30^ ionic lock, the Trp^6.48^ toggle switch and the hydrogen interactions between Tyr^5.58^–Tyr^7.53^.

## Abstract 2

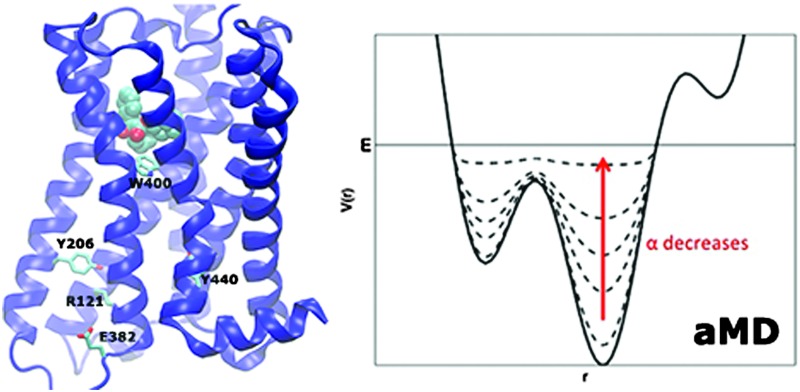
G-protein coupled receptors (GPCRs) mediate cellular responses to various hormones and neurotransmitters and are important targets for treating a wide spectrum of diseases.

## Introduction

G-protein coupled receptors (GPCRs) represent the largest superfamily of membrane proteins that mediate cellular responses to hormones, neurotransmitters, and the senses of sight, olfaction and taste. Due to their critical roles in both the central and parasympathetic nervous systems, GPCRs have served as targets of ∼30–40% of currently marketed drugs for treating a wide spectrum of diseases including cancer, heart failure, asthma, schizophrenia, Alzheimer's and Parkinson's diseases.^1,2^

Most GPCRs have been found to be constitutively active, *i.e.*, they exhibit a certain level of basal activity even without binding an agonist, a molecule that initiates a physiological response.^3^ This suggests that there exists an ensemble of different conformational states (*e.g.*, inactive, intermediate and active) in GPCRs. The conformational equilibrium is biased towards an active state when the receptors are bound by agonists. In contrast, the receptors are switched to an inactive state upon binding of inverse agonists. Additionally, they are able to bind neutral antagonists that have no signaling effects but block binding of other ligands, as well as partial agonists that induce only submaximal activity.^3^

Upon binding of extracellular ligands, the constitutively active GPCRs are able to select conformations for coupling with different intracellular proteins (*e.g.*, the G proteins, arrestins, kinases and phosphorylases) and induce distinct downstream signaling processes. As such ligand-induced allosteric signaling involves highly dynamic conformational selection, the free energy landscape^4^ has been suggested as a tool to study structure, dynamics and function of GPCRs.^5^ The funnel-shaped free energy landscape theory was developed to describe protein folding.^4,6^ It has also been found applicable to protein binding^7^ and many other biological processes that involve population shift of different conformational states, *e.g.*, GPCR allosteric signaling.

The GPCR X-ray structures have been mostly determined in an inactive state,^8^ including the M_2_ and M_3_ muscarinic receptors,^9,10^ the β_2_-adrenergic receptor (β_2_AR),^11^ dopamine D_3_ receptor,^12^ histamine H_1_ receptor,^13^ rhodopsin,^14^ *etc.* Currently, X-ray studies have revealed active structures for two GPCRs, opsin (activated rhodopsin)^15,16^ and β_2_AR coupled with the G_s_-protein or its mimetic nanobody.^17,18^ These structures are characterized by rearrangements of the transmembrane (TM) helices 5, 6 and 7 relative to the inactive configuration, particularly outward tilting of the cytoplasmic end of TM6, breaking of the salt bridge between Arg^3.50^–Glu^6.30^ (the so-called “ionic lock”) and conformational change of the Trp^6.48^ toggle switch.^19,20^ Note that the residue superscripts denote Ballesteros–Weinstein numbering, a convention used to compare across subfamilies of GPCRs.^21^

Computational simulations have been performed to study the conformational states and structural dynamics of GPCRs.^22–28^ Recent microsecond-timescale conventional molecular dynamics (cMD) simulations using the specialized supercomputer “Anton” revealed distinct conformations of the ionic lock (broken and salt-bridged) of the inactive antagonist-bound β_2_AR.^29^ More simulations of unbound β_2_AR identified three conformations in the ionic lock, locked, semi-open with a bridging water molecule and fully open.^30^ Starting from the active X-ray structure, the deactivation of β_2_AR was also modeled upon removal of the G-protein or G-protein-mimetic nanobody and an intermediate was identified during the transition.^25^ Moreover, cMD simulations of the serotonin 2A receptor (5-HT_2A_R) revealed distinct conformational changes in the GPCR activation-associated elements upon binding of the full, partial and inverse agonists.^28^

Anton simulations of the M2 muscarinic receptor showed the inactive X-ray structure bound by antagonist 3-quinuclidinyl-benzilate (QNB) does not undergo significant structural changes during 16.4 μs. More simulations of the apo M2 receptor with antagonist placed in the bulk solvent captured binding to an extracellular vestibule, but not to the orthosteric site.^10^ The apo M2 receptor remained inactive through these simulations.^19^ Thus, longer simulations are desirable for GPCRs and other membrane proteins.^31–33^ Activation that was shown experimentally to occur on millisecond timescales^34^ has not been observed in the longest cMD simulations.^25^

Enhanced sampling techniques have been used to calculate their free energy landscapes,^23^ including adaptive biasing force (ABF),^27,35^ metadynamics,^36^ and simulations combining coarse-grained conformational sampling and cMD.^26,37^ For a set of ligands including agonists, neutral antagonists and inverse agonists, ABF simulations were performed on β_2_AR to investigate the shift of the receptor conformational equilibrium and examine changes in the receptor free energy landscape upon binding of different ligands.^27,35^ The ionic lock distance and side chain dihedral angle *χ*_1_ of the Trp^6.48^ toggle switch were used as two reaction coordinates for calculating the free energies. Calculation results showed that the conformation of β_2_AR is shifted towards the active state by binding full agonist epinephrine, compared with weak partial agonists catechol/dopamine and inverse agonists. In another study of the human adenosine A_2A_ receptor (A_2A_AR),^36^ 500 ns cMD and 100 ns metadynamics simulations were performed on the receptor bound by six different ligands. Using side chain dihedrals *χ*_1_ and *χ*_2_ of the Trp^6.48^ toggle switch as two reaction coordinates, the authors found six conformational states of this key residue. These enhanced sampling studies provide important insights into the free energy landscapes of GPCRs.

One drawback of many enhanced sampling techniques is the requirement of pre-defined reaction coordinates. In comparison, accelerated molecular dynamics (aMD) is another enhanced sampling method with no such requirement. In its simplest form, the algorithm works by adding a non-negative boost potential to the biomolecular potential energy surface, effectively decreasing the energy barriers and thus accelerating transitions between the low-energy states.^38–40^ AMD has been successfully applied to a number of systems^41–45^ and hundreds-of-nanosecond aMD simulations have been shown to capture millisecond-timescale events.^19,46^ Application of aMD enhanced sampling to GPCRs is particularly desirable in order to access long timescales of the dynamic behavior of membrane proteins in the lipid phase.^32,33^

**Fig. 1:**
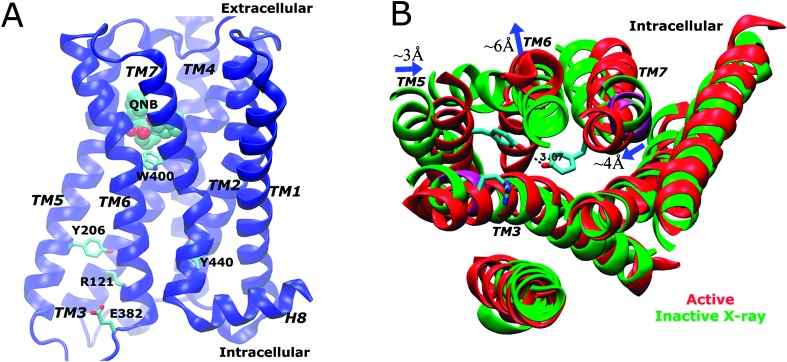
(A) Schematic representation of the X-ray structure of the QNB-bound M2 muscarinic receptor. The protein is rendered as ribbons, the QNB ligand in spheres and key residues Trp400^6.48^ (toggle switch), Arg121^3.50^, Glu382^6.30^, Tyr206^5.58^ and Tyr440^7.53^ in sticks. (B) In comparison with the inactive X-ray structure (green), the active M2 receptor (red) is characterized by breaking of the Arg121^3.50^–Glu382^6.30^ ionic lock, formation of a hydrogen bond between Tyr206^5.58^–Tyr440^7.53^ and outward tilting of the TM6 cytoplasmic end by ∼6 Å (figure adapted from ref. 19).

In a recent study, we applied aMD to simulate the M2 muscarinic receptor and observed its activation in a ligand-free form ref. 19. Starting from the inactive X-ray conformation, the receptor is activated *via* an intermediate state, for which two low-energy conformers with different Tyr206^5.58^ orientations were identified. The active state is characterized by formation of a Tyr206^5.58^–Tyr440^7.53^ hydrogen bond in the intracellular G-protein coupling site and outward tilting of the TM6 cytoplasmic end by ∼6 Å (Fig. 1B). Therefore, aMD simulation enables detailed analysis of the receptor activation pathway and allosteric network at an atomistic level.^19^

Here, the free energy landscape of GPCRs is explored using aMD, as demonstrated on the M2 muscarinic receptor. Previous dual-boost aMD simulations of the antagonist QNB-bound and apo M2 receptor^19^ and a 16.4 μs Anton cMD simulation of the QNB-bound complex provided by D. E. Shaw research are used for free energy calculations in the present study.^10^ Mechanistic differences between the two receptor forms are examined with respect to several key GPCR structural motifs (Fig. 1): the Arg121^3.50^–Glu382^6.30^ ionic lock, Trp400^6.48^ toggle switch, and the Tyr206^5.58^–Tyr440^7.53^ interaction that forms a hydrogen bond in the receptor active state. The calculations highlight the larger conformational space sampled in the apo receptor than in the antagonist-bound form and the population shift of receptor conformations upon ligand binding. Challenges of using aMD enhanced sampling for free energy calculations of GPCRs, including energetic reweighting, are also discussed.

## Methods

### Accelerated molecular dynamics

**Fig. 2:**
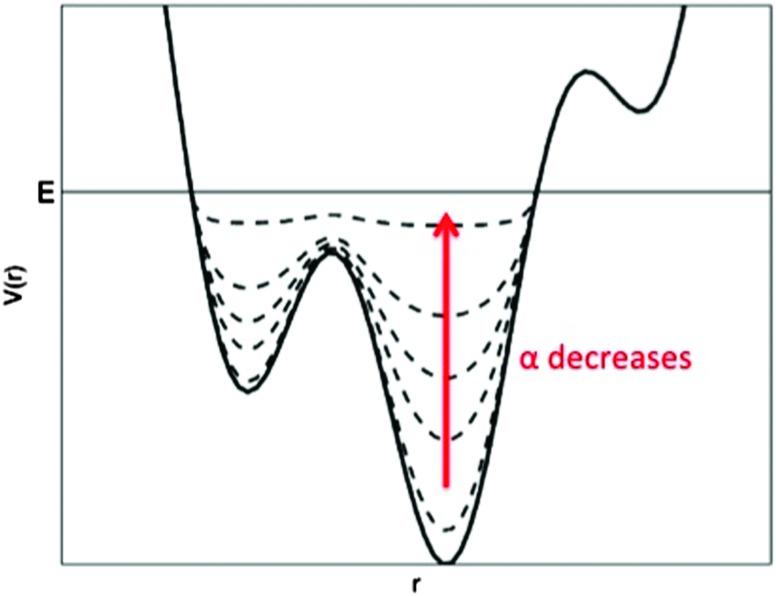
In the simplest form of accelerated molecular dynamics, non-negative boost potential is added to biomolecular potential surface when the system potential is lower than a reference energy *E*. As the acceleration factor *α* decreases, the potential energy surface is flattened more and transitions between different low-energy states become increased.

AMD enhances the conformational sampling of biomolecules by adding a non-negative boost potential to the potential energy surface when the system potential is lower than a reference energy:^38–40^ V*(*r*) = *V*(*r*), *V*(*r*) ≥ *E*,

1 V*(*r*) = *V*(*r*) + Δ*V*(*r*), *V*(*r*) < *E*,

where *V*(*r*) is the original potential, *E* is the reference energy, and V*(*r*) is the modified potential. The boost potential, Δ*V*(*r*) is given by:2
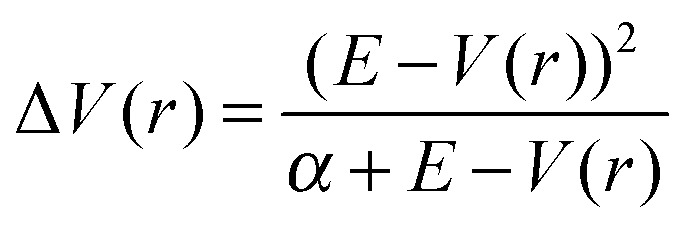
where *α* is the acceleration factor. As the acceleration factor *α* decreases, the potential energy surface is flattened and biomolecular transitions between the low-energy states are increased as illustrated in Fig. 2.

Two versions of aMD that provide different acceleration schemes have been developed, *i.e.*, dihedral-boost^39^ and dual-boost.^40^ In dihedral-boost aMD, the bias potential is applied to all dihedral angles in the system with input parameters (*E*_dihed_, *α*_dihed_). In dual-boost aMD, a total boost potential is applied to all atoms in the system in addition to the dihedral boost, *i.e.*, (*E*_dihed_, *α*_dihed_; *E*_total_, *α*_total_). For simulations of membrane proteins such as GPCRs, the input parameters can take the following form:^19^ *E*_dihed_ = *V*_dihed_avg_ + *λ* × *V*_dihed_avg_, *α*_dihed_ = *λ* × *V*_dihed_avg_/5

3 *E*_total_ = *V*_total_avg_ + 0.2 × *N*_atoms_, *α*_total_ = 0.2 × *N*_atoms_,

where *N*_atoms_ is the total number of atoms, *V*_dihed avg_ and *V*_total avg_ are the average dihedral and total potential energies calculated from short cMD simulations, respectively, and *λ* is an adjustable acceleration parameter. Previous study suggested proper acceleration is achieved at *λ* = 0.3 for simulation of membrane proteins.^19^ The usage of *λ* × *V*_dihed avg_, instead of the number of residues as implemented in earlier aMD simulations of soluble proteins,^39,47^ is applied to account for the very different number of dihedrals in lipid molecules and protein amino acid residues.

### AMD simulation of the M2 muscarinic receptor

Simulation of the M2 muscarinic receptor was carried out using the co-crystallized X-ray structure of the QNB–M2 complex (PDB: 3UON) that was solved at 3.0 Å resolution.^9^ The simulation details have been described in a previous study^19^ and a brief summary will be provided here. The T4 lysozyme chimera that replaced intracellular loop 3 (ICL3) to facilitate receptor crystallization was omitted, the chain termini were capped with neutral groups (acetyl and methylamide), and the two disulphide bonds Cys96^3.25^–Cys176^ECL2^ and Cys413^6.61^–Cys416^7.29^ were maintained. Protein residues were set to the standard CHARMM protonation states at neutral pH, with the exception of Asp69^2.50^ which is buried in the hydrophobic core and thus protonated.^10^ The M2 receptor was then embedded in a palmitoyl-oleoyl-phosphatidyl-choline (POPC) lipid bilayer and solvated an aqueous medium of 0.15 M NaCl with all atoms represented explicitly for all the simulations. The QNB ligand was removed from the orthosteric site to simulate the M2 receptor in the apo form. For this membrane–protein complex system, the CHARMM27 parameter set with CMAP terms included was used for the protein,^48,49^ CHARMM36 for POPC lipids,^50^ and TIP3P model for water molecules.^51^ Force field parameters for QNB were obtained from the CHARMM ParamChem web server.^52^

AMD simulations were performed using NAMD2.9^53,54^ on both the apo and QNB-bound forms of the M2 receptor by restarting from the final structure of 100 ns cMD simulations. Previous study showed that dihedral-boost aMD simulations did not provide high enough acceleration to achieve sufficient conformational sampling.^19^ Thus, dual-boost aMD simulations are used for the free energy calculations here. The bias potential was applied to all dihedral angles (dihedral-boost) and all individual atoms (total-boost) with the following parameters: *E*_dihed_ = *V*_dihed_avg_ + 0.3 × *V*_dihed_avg_, *α*_dihed_ = 0.3 × *V*_dihed_avg_/5; *E*_total_ = *V*_total_avg_ + 0.2 × *N*_atoms_ and *α*_total_ = 0.2 × *N*_atoms_, where *N*_atoms_ is the total number of atoms and *V*_dihed_avg_ and *V*_total_avg_ are the average dihedral and total potential energies calculated from the 100 ns cMD simulations, respectively. Five production aMD simulations were obtained on the apo M2 receptor, *i.e.*, one for 400 ns and four for 200 ns, and one production aMD run on the QNB-bound form for 200 ns.

### Free energy calculation

The potential of mean force (PMF), used synonymously with free energy profile in the literature, examines how the free energy changes as a function of specific reaction coordinates. PMF profiles of the M2 muscarinic receptor were calculated for a set of reaction coordinates that have been adopted to characterize GPCR activation, including the ionic lock distance between charge centers of the Arg121^3.50^ (C_ζ_ atom) and Glu382^6.30^ (C_δ_ atom) side chains, two side chain dihedral angles *χ*_1_ and *χ*_2_ in the Trp400^6.48^ toggle switch and the distance between hydroxyl oxygen atoms of the Tyr206^5.58^ and Tyr440^7.53^ side chains (Fig. 1A). All PMF calculations were performed using five different bin sizes, *i.e.*, 0.1–0.5 Å for the Arg121^3.50^–Glu382^6.30^ ionic lock and the Tyr206^5.58^–Tyr440^7.53^ hydrogen bond, and 3–15 degrees for side chain dihedrals *χ*_1_ and *χ*_2_ in the Trp400^6.48^ toggle switch.

Five production dual-boost aMD simulations of the apo M2 receptor (one for 400 ns and four for 200 ns) were combined into one trajectory with a total length of 1200 ns for calculating the free energy profiles and one 200 ns production aMD simulation for the QNB-bound form. Snapshots were taken every 1 ps from the aMD simulations for data collection. PMF profiles of the QNB-bound form in aMD simulation are compared with those of the 16.4 μs Anton cMD simulation provided by D. E. Shaw research,^10^ for which data were collected at 180 ps intervals based on the trajectory frames saved by the authors.

**Fig. 3:**
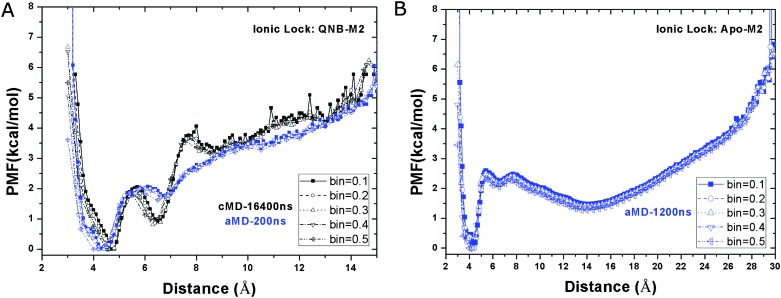
Free energy profiles of the ionic lock distance between Arg121^3.50^–Glu382^6.30^ in aMD simulations of the (A) QNB-bound and (B) apo forms of the M2 receptor. Five different bin sizes 0.1–0.5 Å are used. The PMF profiles calculated from the 16.4 μs Anton cMD simulation of the QNB-bound form are also plotted for comparison in (A).

In principle, free energy profiles of aMD simulations can be reweighted using the Boltzmann factor of the boost potential applied to each trajectory frame, *i.e.*, e^Δ*V*(*r*)/*k*_B_*T*^, where *k*_B_ is the Boltzmann's constant and *T* is the temperature.^39^ However, due to large energetic noise observed in reweighting of the M2 receptor aMD simulations (see example reweighted PMF profiles for the Arg121^3.50^–Glu382^6.30^ ionic lock in the QNB-bound form in ESI†), unweighted free energy profiles are presented in this study. Bear in mind that transition barriers between low-energy states are decreased in aMD-sampled PMF profiles, but the overall shape of the free energy profiles shall be maintained from the original as illustrated in Fig. 2. In the absence of reweighting, comparison of PMF profiles obtained from the aMD and Anton cMD simulations (*e.g.*, Fig. 3A for the ionic lock in the QNB-bound form) also provides an estimate of the free energy differences.

## Results and discussion

**Fig. 4:**
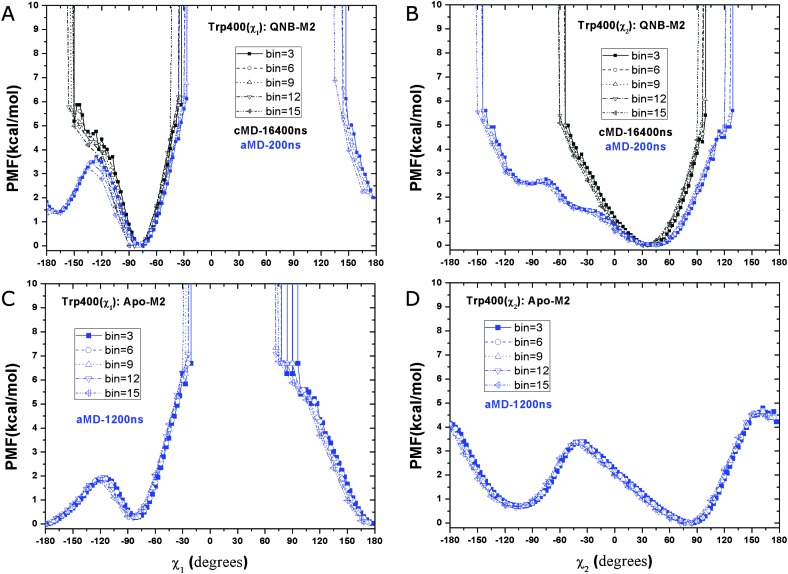
Free energy profiles of two side chain dihedral angles in the Trp400^6.48^ toggle switch in aMD simulations of the M2 receptor: (A) *χ*_1_ and (B) *χ*_2_ in the QNB-bound form, and the two dihedrals in the apo form in (C) and (D), respectively. Five different bin sizes 3–15 degrees are used. Similar to Fig. 3, PMFs of the QNB-bound form in the 16.4 μs Anton simulation are also shown in (A) and (B).

Free energy profiles of the M2 muscarinic receptor are explored using aMD simulations in terms of the receptor ionic lock formation, the toggle switch sampling and hydrogen bonding interactions between two intracellular tyrosine residues, all of which have been noted previously to be important for GPCR activation. Specifically, PMF profiles are calculated for the ionic lock distance between Arg121^3.50^–Glu382^6.30^, side chain dihedral angles *χ*_1_ and *χ*_2_ in the Trp400^6.48^ toggle switch, and the interaction distance between Tyr206^5.58^–Tyr440^7.53^ (see details in Methods). Five different bin sizes were used for the PMF calculations to estimate their precision. In the case of poor sampling, the calculated PMF profiles will exhibit large variations at different bin sizes. However, negligible differences are observed in the presented PMF profiles at various bin sizes (Fig. 3, 4 and 6). This indicates convergent sampling of the distance and dihedral reaction coordinates are achieved and thus precise PMF profiles are obtained.^55^

In aMD simulation of the apo form, the M2 receptor samples multiple conformational states including inactive, intermediate and active. Upon binding of antagonist QNB in the orthosteric site, the receptor conformation is biased towards the inactive state with fewer conformations visited by the key structural motifs. PMF profiles of the QNB-bound M2 receptor obtained from aMD simulation are also compared with those of the 16.4 μs Anton cMD simulation provided by D. E. Shaw research.^10^ Below we will discuss each of the free energy profiles in detail.

### Ionic lock

In the QNB-bound M2 receptor, a relative free energy minimum of the Arg121^3.50^–Glu382^6.30^ ionic lock is observed at ∼4.2 Å distance in the 200 ns aMD simulation (Fig. 3A). A second energy minimum is found at ∼6.8 Å. In the 16.4 μs Anton cMD simulation,^10^ two similar energy minima are observed at 4.6 Å and 6.4 Å, respectively. The second energy well appears to be shallower in aMD than in the Anton cMD simulation, likely due to the unweighted PMF profile obtained from aMD simulation. These findings show that the Arg121^3.50^–Glu382^6.30^ ionic lock adopts two distinct conformations in the QNB-bound M2 receptor, consistent with previous microsecond-timescale cMD simulation of β_2_AR.^29^

In aMD simulation of the apo M2 receptor, while the relative free energy minimum of the ionic lock remains at ∼4.6 Å, the second energy well centered at 6.4 Å is less populated (Fig. 3B). Additionally, a third energy well appears with broad distribution centered at ∼14.2 Å and a minimum at approximately 1.5 kcal mol^–1^ above the zero energy minimum. This long Arg121^3.50^–Glu382^6.30^ residue distance indicates a large separation of the TM6 cytoplasmic end from TM3 in the activated state of the receptor.^19^

In the ligand-free form of the M2 receptor, the GPCR ionic lock located in the intracellular G-protein-coupling site samples multiple conformational states. These states correspond to the closed (locked), semi-open with a bridging water molecule and fully open conformations as discussed in earlier study of the apo β_2_AR.^30^ Binding of ligands in the orthosteric site may shift this conformational equilibrium to one with fewer states. For example, the antagonist QNB appears to stabilize the ionic lock and confine its motion to the closed and water-bridged conformations, by which the M2 receptor stays in an inactive state.

### Toggle switch

In the 16.4 μs Anton simulation of the inactive QNB-bound M2 receptor, side chain dihedral angles *χ*_1_ and *χ*_2_ of the Trp400^6.48^ toggle switch are confined in energy wells with minima found at –75° (Fig. 4A) and 40° (Fig. 4B), respectively. In the 200 ns aMD simulation, *χ*_1_ is able to visit another energy well at –165 degrees and the minimum is ∼1.5 kcal mol^–1^ higher than the zero free energy. The barrier of transition from relative energy minimum to the second energy well is nearly 4 kcal mol^–1^ (Fig. 4A). For *χ*_2_ of the QNB-bound form, the free energy well centered at 40° becomes significantly wider in aMD than in the 16.4 μs Anton simulation, suggesting a larger sampled conformational space as shown in Fig. 4B.

In the aMD simulation of the apo M2 receptor, *χ*_1_ largely shifts its population to the ±180° energy minimum well. This second energy well is slightly more populated than the one centered at –75° that is dominant in the QNB-bound form (Fig. 4C). Similarly, *χ*_2_ visits a second energy well centered at –105° and it is separated from the relative energy minimum by ∼3.5 kcal mol^–1^ transition barrier (Fig. 4B). Hence, the toggle switch in the apo form clearly samples more conformations than in the QNB-bound form.

**Fig. 5:**
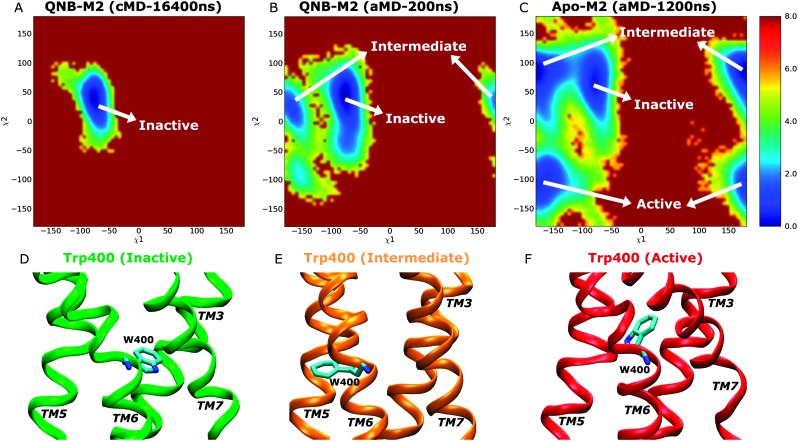
Two dimensional free energy profiles of side chain dihedrals *χ*_1_ and *χ*_2_ in the Trp400^6.48^ toggle switch of the M2 receptor: the QNB-bound form of (A) 16.4 μs Anton simulation and (B) 200 ns aMD simulation, and (C) the apo form of a total length of 1200 ns aMD simulation. Calculated results using a bin size of 6 degrees are shown here. Representative conformations of the Trp400^6.48^ toggle switch sampled in aMD simulation: (D) inactive, (E) intermediate and (F) active.

Two dimensional free energy profiles of dihedral angles (*χ*_1_, *χ*_2_) in the Trp400^6.48^ toggle switch are shown in Fig. 5. In the Anton simulation of the QNB-bound form, only one energy well is identified, with minimum at (–75°, 40°) (Fig. 5A). This well corresponds to an inactive conformation of the toggle switch as shown in Fig. 5D. In contrast, the toggle switch in biased aMD simulation of the QNB-bound form visits another two conformations with minima found at (–165°, 40°) (intermediate) and (–160°, –100°) (active), respectively, although the latter two are much less populated with higher free energies, *i.e.*, ∼2 kcal mol^–1^ and 3 kcal mol^–1^ (Fig. 5B). Apo aMD simulation of the constituently active M2 receptor sample significantly larger conformational space with respect to the toggle switch, including a higher population found in the intermediate and active state conformations (Fig. 5C). The intermediate conformation depicts translocation of the Trp400^6.48^ side chain towards TM5 (Fig. 5E). The active toggle switch exhibits tilting of the Trp400^6.48^ side chain towards the ligand-binding pocket (Fig. 5F), which is similar to the active conformation observed in opsin^15,16^ and the active β_2_AR,^17,18^ as well as the agonist-bound A_2A_AR.^20,36^ Such observations agree well with the hypothesis that conformational changes in the toggle switch play a key role in activation of GPCRs.

### Interactions between Tyr206^5.58^–Tyr440^7.53^

**Fig. 6:**
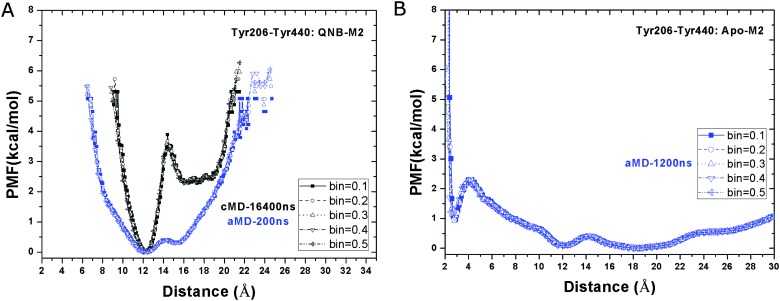
Free energy profiles of the distance between the hydroxyl oxygens of the Tyr206^5.58^–Tyr440^7.53^ side chains in aMD simulations of the (A) QNB-bound and (B) apo forms of the M2 receptor. Five different bin sizes 0.1–0.5 Å are used. Similar to Fig. 3, PMFs of the QNB-bound form in the 16.4 μs Anton simulation are also shown in (A).

Interactions between two intracellular tyrosine residues, Tyr206^5.58^ and Tyr440^7.53^, are thought to be relevant for GPCR activation and their conformations are closely examined as follows. In the inactive X-ray structure of the QNB-bound M2 receptor, the distance between Tyr206^5.58^ and Tyr440^7.53^ hydroxyl oxygen atoms is 12.6 Å.^9^ As shown in Fig. 6A, this distance is consistently found to be the relative free energy minimum in both the Anton cMD and aMD simulations of the QNB-bound receptor. A second energy well with a minimum found at larger distance, *i.e.*, ∼16–20 Å and ∼16 Å in the Anton and aMD simulations, respectively, which potentially represents a more open conformation between the two tyrosine residues. Notably, the second energy well is separated from the relative energy minimum by ∼4 kcal mol^–1^ transition barrier in the Anton simulation. As expected, lower energy barrier is found in the unweighted aMD PMF profiles. In all simulations of the inactive QNB-bound M2 receptor, these two tyrosine residues do not sample a hydrogen-bonded conformation.

In the apo aMD simulation, the Tyr206^5.58^–Tyr440^7.53^ residue pair form a hydrogen bond, populating a third energy well with minimum found at 2.6 Å (Fig. 6B). In addition, the receptor conformation is shifted more towards the energy well with broad distribution at ∼16–20 Å, which becomes slightly more favorable than the 12.6 Å energy minimum found in simulations of the QNB-bound form.

From these diverse simulations it is clear that the distance between hydroxyl oxygens of the Tyr206^5.58^–Tyr440^7.53^ side chains can sample fully open, semi-open and closed (hydrogen bonded) conformations, which correspond to the inactive, intermediate and active states of the receptor, respectively. It is also worth noting from our previous study that the formation of this hydrogen bond is strongly correlated with breaking of the Arg121^3.50^–Glu382^6.30^ ionic lock during activation of the M2 receptor.^19^

## Conclusions

Recent years have seen an explosion of new high-resolution X-ray structures of GPCRs,^56,57^ notably the active opsin^15^ and β_2_AR^11^ that are coupled with the C-terminal peptide of the G_α_ subunit and the heterotrimeric G_s_ protein, respectively. Despite such tremendous advances, X-ray structures represent only static snapshots of GPCRs during cell signaling processes. Detail functional mechanisms of this important family of membrane proteins remain unclear.

GPCRs are able to interact with different intracellular proteins, including the G proteins, arrestins, kinases and phosphorylases. They also respond to a wide variety of extracellular stimuli, such as light, hormones, neurotransmitters, odorants, lipids, amino acids, nucleotides and chemokines. Binding of pharmacologically different ligands (*e.g.*, antagonists, inverse agonists, full and partial agonists) shifts the population of constitutively active GPCR conformations and induces distinct signaling pathways. Hence, free energy landscapes are useful for describing the structural dynamics and allosteric signaling of GPCRs.^5^

In the present study, the free energy profiles of the M2 muscarinic receptor are explored through aMD enhanced sampling. Hundreds-of-nanoseconds dual-boost aMD simulations have been shown to capture activation of the M2 receptor on the millisecond timescales.^19^ PMF profiles are calculated for the Arg121^3.50^–Glu382^6.30^ ionic lock, Trp400^6.48^ toggle switch, and the Tyr206^5.58^–Tyr440^7.53^ hydrogen bond partners in apo and QNB-bound forms of the M2 receptor, and they indicate differences of the two receptor forms in sampling active, intermediate and inactive states.

In the apo form, the M2 receptor generally samples multiple conformations in the activation-associated structural motifs. Notably, both the Arg121^3.50^–Glu382^6.30^ ionic lock and the Tyr206^5.58^–Tyr440^7.53^ hydrogen bond partners exhibit three closed, semi-open and fully open conformations. The formation of the Tyr206^5.58^–Tyr440^7.53^ hydrogen bond (open-to-closed transition) is strongly correlated with breaking of the ionic lock (closed-to-open transition) during activation of the receptor. Furthermore, two dimensional free energy profiles of (*χ*_1_, *χ*_2_) in Trp400^6.48^ also showed that the toggle switch samples three conformations, corresponding to the inactive, intermediate and active states of the M2 receptor.

Upon binding of the QNB antagonist, the M2 receptor significantly shifts the population of conformations on the free energy landscape and the structural motifs sample fewer conformations. The X-ray structure of the QNB-bound M2 receptor consistently corresponds to energy minimum conformations revealed from the Anton and aMD simulations, with exception of the ionic lock. The ionic lock is closed in the simulation-derived energy minimum conformation, but open in the X-ray structure. One explanation for this discrepancy may be the effect of replacing the third intracellular loop with T4-lysozyme for crystallization. It has been suggested that the presence of lysozyme significantly destabilizes the ionic lock.^29^

GPCR cellular signaling involves ligand binding, G-protein coupling, nucleotide exchange in the activated G-protein and allosteric regulation, as well as oligomerization. These processes occur on the millisecond timescales and even longer which cannot be accessed by current unbiased simulation protocols. As demonstrated on activation of the M2 muscarinic receptor, aMD is a promising enhanced sampling technique that can be used to simulate these processes and explore GPCR free energy landscapes in future studies of these areas.

Remaining challenges include particularly simulating the GPCR-G protein complex with increasing system size and complexity^58^ and suppressing aMD energetic noise to recover the canonical ensemble and thus original free energy landscape of large biomolecules.^38^ Cumulant expansion is found to greatly improve energetic reweighting of aMD simulations on alanine dipeptide and fast-folding proteins.^59,60^ With increasing system size, large boost potential with broad distribution (*i.e.*, large standard deviation) is applied to the system, which leads to high fluctuations in the reweighted free energy profiles. Nevertheless, selectively applied aMD simulations,^61^ replica-exchange aMD simulations^62–64^ and population-based reweighting of scaled MD simulations^65^ have been developed to facilitate biomolecular free energy calculations. Other techniques that apply reduced boost potential while maintaining sufficient sampling can also be helpful for energetic reweighting of aMD simulations for exploring GPCR free energy landscapes.
