# Supplementary material for: Free energy landscape of G-protein coupled receptors, explored by accelerated molecular dynamics
Source: Phys Chem Chem Phys. 2014 Jan 21;16(14):6398–406. doi: 10.1039/c3cp53962h (PMC3960983; doi:10.1039/c3cp53962h)
Supplement: Supplementary file 1 [file CP-016-C3CP53962H-s001.pdf]

Supporting Information

for "Free Energy Landscape of G-Protein Coupled Receptors, Explored by Accelerated Molecular Dynamics" by Yinglong Miao, Sara E. Nichols and J. Andrew McCammon

Reweighting of aMD simulations

Reweighting Methods

Details of aMD reweighting methods are described in Ref. 1 and a summary is provided here. For aMD simulation of a biomolecular system, the probability distribution along a selected reaction coordinate $A(\boldsymbol{r})$ is written as $p^*(A)$, where $\boldsymbol{r}$ denotes the atomic positions $\{\boldsymbol{r}_1,\cdots,\boldsymbol{r}_N\}$. Given the boost potential $\Delta V(\boldsymbol{r})$ of each frame, $p^*(A)$ can be reweighted to recover the canonical ensemble distribution, $p(A)$, as:

$$p(A_j)=p^*(A_j)\frac{\left\langle e^{\beta\Delta V(\boldsymbol{r})}\right\rangle_j}{\sum_{j=1}^{M}\left\langle e^{\beta\Delta V(\boldsymbol{r})}\right\rangle_j},\ j=1,\cdots,M, \tag{S1}$$

where M is the number of bins and $\left\langle e^{\beta\Delta V(\boldsymbol{r})}\right\rangle_j$ is the ensemble-averaged Boltzmann factor of $\Delta V(\boldsymbol{r})$ for simulation frames found in the jth bin. The above equation provides an "exponential average" algorithm for aMD reweighting of aMD simulations. The reweighted potential of mean force (PMF) is calculated as $F(A_j)=-\frac{1}{\beta}\ln p(A_j)$.

As the Boltzmann factors are often dominated by high boost potential frames that are poorly sampled, the aMD reweighting based on exponential average generally leads to high energetic fluctuations[1, 2]. To reduce the energetic noise, the exponential term can be approximated as summation of the Maclaurin series of boost potential $\Delta V(\boldsymbol{r})$ and reweighting factor is rewritten as:

$$\left\langle e^{\beta\Delta V}\right\rangle=\sum_{k=0}^{\infty}\frac{\beta^k}{k!}\left\langle \Delta V^k\right\rangle \tag{S2}$$

where the subscript *j* has been suppressed. The Maclaurin series expansion up to the 5th-10th order has been used in practice to reweight aMD trajectories[3]. The reweighted PMF profiles are typically less noisy than those obtained from exponential average reweighting.

Furthermore, the ensemble-averaged reweighting factor can be approximated using cumulant expansion[4, 5]:

$$\left\langle e^{\beta \Delta V} \right\rangle = \exp\left\{ \sum_{k=1}^{\infty} \frac{\beta^k}{k!} C_k \right\}, \qquad \text{(S3)}$$

where the first three cumulants are given by:

$$\begin{aligned} C_1 &= \left\langle \Delta V \right\rangle, \\ C_2 &= \left\langle \Delta V^2 \right\rangle - \left\langle \Delta V \right\rangle^2 = \sigma_{\Delta V}^2, \\ C_3 &= \left\langle \Delta V^3 \right\rangle - 3\left\langle \Delta V^2 \right\rangle \left\langle \Delta V \right\rangle + 2\left\langle \Delta V \right\rangle^3. \end{aligned} \qquad \text{(S4)}$$

Note that the Maclaruin series expansion is equivalent to cumulant expansion on the 1st order[1]:

$$\left\langle e^{\beta \Delta V} \right\rangle = \sum_{k=0}^{\infty} \frac{\beta^k}{k!} \left\langle \Delta V^k \right\rangle = e^{\beta \langle \Delta V \rangle}. \qquad \text{(S5)}$$

Reweighted free energy profiles

Based on the exponential average and cumulant expansion reweighting methods, PMF profiles are calculated for the Arg121$^{3.50}$-Glu382$^{6.30}$ ionic lock in the QNB-bound M2 receptor as shown in **Fig. S1**. Compared with the PMF profile obtained from the 16.4 μs Anton simulation, large energetic noise is observed in the reweighted PMF profiles, particularly using exponential average and cumulant expansion to the 2nd order. Cumulant expansion on the 1st order (equivalent to the Maclaurin series expansion) reduces the energetic noise, but the reweighted PMF provides incorrect energy minimum positions and significant errors as seen in **Fig. S1B**.

While cumulant expansion to the 2nd order is found to greatly improve energetic reweighting of aMD simulations on alanine dipeptide and fast-folding proteins[1], for large proteins like the M2 muscarinic receptor considered here, high boost potential with broad distribution in the range of ~200 kcal/mol is applied. This leads to high fluctuations in the reweighted free energy profiles of the M2 receptor, and thus the unweighted profiles are presented in the text.

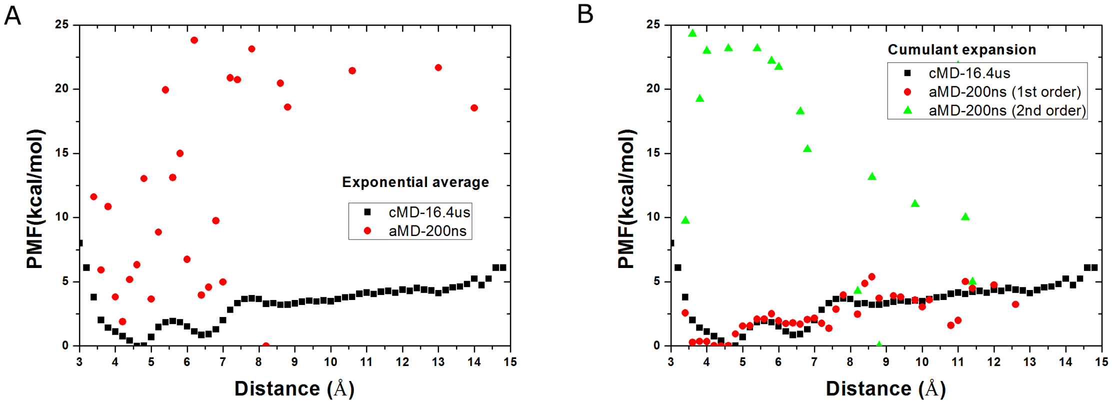

Fig. S1 PMF profiles of the Arg121$^{3.50}$-Glu382$^{6.30}$ ionic lock in the QNB-bound M2 receptor obtained from reweighting based on (A) exponential average and (B) cumulant expansion to the 1st order (equivalent to Maclaurin series expansion) and 2nd order. PMF calculated from the 16.4 μs Anton simulation is also plotted for comparison.

References

1. Y. Miao, W. Sinko, L. Pierce, D. Bucher and J. A. McCammon, *In preparation*, 2013.
2. T. Y. Shen and D. Hamelberg, *J Chem Phys*, 2008, **129**.
3. L. C. T. Pierce, R. Salomon-Ferrer, C. A. F. de Oliveira, J. A. McCammon and R. C. Walker, *J Chem Theory Comput*, 2012, **8**, 2997-3002.
4. G. Hummer, *J Chem Phys*, 2001, **114**, 7330-7337.
5. M. P. Eastwood, C. Hardin, Z. Luthey-Schulten and P. G. Wolynes, *J Chem Phys*, 2002, **117**, 4602-4615.
